# Rescue of Inhibitory Synapse Strength following Developmental Hearing Loss

**DOI:** 10.1371/journal.pone.0053438

**Published:** 2013-01-11

**Authors:** Vibhakar C. Kotak, Anne E. Takesian, Patricia C. MacKenzie, Dan H. Sanes

**Affiliations:** 1 Center for Neural Science, New York University, New York, New York, United States of America; 2 Department of Biology, New York University, New York, New York, United States of America; University of Cincinnatti, United States of America

## Abstract

Inhibitory synapse dysfunction may contribute to many developmental brain disorders, including the secondary consequences of sensory deprivation. In fact, developmental hearing loss leads to a profound reduction in the strength of inhibitory postsynaptic currents (IPSCs) in the auditory cortex, and this deficit persists into adulthood. This finding is consistent with the general theory that the emergence of mature synaptic properties requires activity during development. Therefore, we tested the prediction that inhibitory strength can be restored following developmental hearing loss by boosting GABAergic transmission *in vivo*. Conductive or sensorineural hearing loss was induced surgically in gerbils prior to hearing onset and GABA agonists were then administered for one week. IPSCs were subsequently recorded from pyramidal neurons in a thalamocortical brain slice preparation. Administration of either a GABA_A_ receptor a1 subunit specific agonist (zolpidem), or a selective GABA reuptake inhibitor (SGRI), rescued IPSC amplitude in hearing loss animals. Furthermore, this restoration persisted in adults, long after drug treatment ended. In contrast, a GABA_B_ receptor agonist baclofen did not restore inhibitory strength. IPSCs could also be restored when SGRI administration began 3 weeks after sensory deprivation. Together, these results demonstrate long-lasting restoration of cortical inhibitory strength in the absence of normal experience. This suggests that *in vivo* GABA_A_ receptor activation is sufficient to promote maturation, and this principle may extend to other developmental disorders associated with diminished inhibitory function.

## Introduction

An influential theory in developmental plasticity states that inhibitory synapse function regulates the excitatory synapse critical period [1], [2], [3], [4]. Support for this idea comes from experiments in which the critical period for ocular dominance plasticity (i.e., the reduced cortical activation by a deprived eye) is accelerated or terminated prematurely by agents that augment GABA_A_ receptor-mediated transmission [5], [6], [7]. However, the normal emergence of inhibitory transmission, itself, depends on both spontaneous and driven activity [2], [4], [8]. In the auditory system, developmental deprivation of sound induces a profound reduction of inhibitory strength, and this weakened inhibition persists into adulthood [9], [10], [11], [12], [13], [14]. Here, we evaluate the extent to which cortical inhibitory synapse maturation relies on GABA receptor activation in animals reared with hearing loss.

A reduction in GABA signaling can delay or prevent the maturation of inhibitory synapses. For example, mice lacking the enzyme associated with GABA synthesis, glutamic acid decarboxylase (GAD) 65, do not exhibit ocular dominance plasticity; however, treatment with benzodiazepines restores this mechanism [15]. Similarly, neuronal cultures from mice deficient in GAD67 display a loss of inhibitory terminals on cortical pyramidal cells, but a normal number of anatomical contacts are restored by pharmacological activation of GABAergic transmission [16]. Thus, we reasoned that interventions employing GABAergic agonists immediately following hearing loss could prevent the functional decline of inhibitory strength, even in the absence of normal hearing.

Experimental approaches to repair brain function by modifying inhibition differ depending upon the goal, and the nature of the disorder. For example, research on ocular dominance plasticity seeks to reduce inhibition in adults in order to restore excitatory synaptic plasticity after the critical period has ended [17], [18], [19]. Other investigators have explored stem cell transplantation to facilitate *de novo* synaptogenesis, and the augmentation of inhibitory drive [20], [21], [22]. Since improved behavioral or neural performance is correlated with stronger GABAergic transmission [23], [24], [25], we sought to reverse the hearing loss-induced dysfunction of inhibitory synaptic strength in the thalamorecipient auditory cortex (ACx).

To test the role of GABA receptor activation, hearing loss was induced prior to hearing onset and GABA agonists were then administered for one week. The amplitude of inhibitory currents was assessed in thalamorecipient ACx neurons in brain slices. Three agents were chosen to activate GABA_A_ receptors only (zolpidem, an a1-subunit potentiator), both GABA_A_ and GABA_B_ receptors (selective GABA reuptake inhibitor, SGRI,1-[2-[[(Diphenylmethylene)imino]oxy]ethyl]-1,2,5,6-tetrahydro-3-pyridinecarboxylic acid hydrochloride hydrochloride and GABA_B_ receptors only (baclofen, BAC). Zolpidem and SGRI restored inhibitory strength while BAC treatment was ineffective. Furthermore, inhibitory restoration persisted in adults. These findings suggest that the basic principle of activity-dependent synapse maturation can be used as a strategy to overcome the deficits that may attend early sensory deprivation.

## Methods

### Animals

Animal care, maintenance, surgery and pharmacological treatments were in accordance with the guidelines and rules of the Institutional Animal Care and Use Committee, New York University (NYU), approved by the Office of Laboratory Animal Welfare, Office of Extramural Research, and U.S. National Institutes of Health (NIH; Bethesda, MD, USA). *See SI Methods* for details.

### Hearing loss surgeries

Gerbil (*Meriones unguiculatus*) pups at postnatal day (P) 10 (P10) were anesthetized with the halogenated ethyl methyl ether methoxyflurane (Metofane). Anesthetic effect occurred by 10 min as tested by an absence of response to nociceptive stimuli (toe pinch). Conductive hearing loss was induced by tympanic membrane puncture and malleus removal. Cochlear ablations were performed on gerbil pups at P10, just prior to the onset of response to airborne sound [10]. *See SI Methods* for details on sham surgery and sham injection.

### *In vivo* pharmacological treatment

A key objective in this study was an attempt to rescue inhibitory synaptic strength following conductive hearing loss. In order to be less invasive and support cortical inhibitory synapses at a slow and sustained level, we subcutaneously administered conductive hearing loss (CHL) pups once everyday for 7 days (i.e. during P11 through P17.) We administered the GABA_A_ receptor a1 subunit-specific agent zolpidem (Sigma, 10 mg/kg), or a selective GABA reuptake inhibitor (SGRI, NO-711 hydrochloride, 1-[2-[[(Diphenylmethylene)imino]oxy]ethyl]-1,2,5,6-tetrahydro-3-pyridinecarboxylic acid, Sigma, 10 mg/kg), or the GABA_B_ agonist BAC (Sigma, 2.5 mg/kg). In a separate group of CHL pups, we administered SGRI (10 mg/kg) during P30–36 following CHL.

The doses were determined based on pilot experiments in which zolpidem, SGRI, or BAC was administered in vivo. The initial does of each drug was 1 mg/kg in 2 CHL animals, and this did not have an apparent effect on spontaneous inhibitory postsynaptic current (sIPSC) amplitudes. The dose was then increased to 10 mg/kg, and this appeared to restore sIPSC amplitude. Therefore, we chose to retain this dose for the study. For BAC, 10 mg/kg led to a decline in motor activity over several days, and we therefore reduced the BAC dosage to 2.5 mg/kg. For a comprehensive outline of the protocol, see Table 1. *See SI Methods* for details.

**Table 1:** Experimental groups.

P10	P11–17	P18–22	P30–36	P90–110
Control		record		
Control				record
CHL		record		
CHL				record
CHL	zolpidem	record		
CHL	SGRI	record		
CHL	baclofen	record		
CHL	zolpidem			record
CHL	SGRI			record
CHL	baclofen			record
CHL			SGRI	record
CHL	sham inject	record		
Ablated	zolpidem	record		
Ablated	zolpidem			record
Sham surgery		record		
Sham surgery	SGRI	Record		

Timing of hearing loss surgery, drug delivery, and brain slice recordings are shown. Conductive hearing loss (CHL) or bilateral cochlear ablation was induced at P10, just prior to hearing onset. Our previous report characterized the effect of CHL on IPSCs at P18–22 and P90–110 (gray box) (14). Following CHL, GABA receptor activators (zolpidem, SGRI or baclofen) were administered subcutaneously once per day for a week (P11–17). Inhibitory currents were recorded from thalamorecipient ACx L2/3 pyramidal neurons at P17–22 or P90–110 (adult) control as well as drug-treated animals. In a separate group of CHL animals, SGRI was administered at a later age (P30–36) and recordings obtained at P90–110. In a final group, zolpidem was administered to SNHL animals and recordings in ACx slices were obtained at P18–22. For controls, sIPSCs, me-IPSCs and PPR were also recorded in juveniles in several additional groups that included sham surgery, sham surgery with SGRI injection and sham injections in CHL animals. N values and animals used for each group are stated in Results, and S1 methods and results.

### Brain slice preparation and whole cell recordings

Thalamocortical brain slices (500 µm) were generated as described in our previous papers [10], [11], [12]. *See S1 Methods* for details on whole-cell recordings and selective stimulation of putative unitary afferents for eliciting minimum evoked (me) IPSCs.

### Statistics

Statistical tests of data distribution were followed by comparisons between groups using commercial software (JMP, SAS Institute). A Shapiro-Wilcoxon W (Goodness-of-fit) test was performed to test for a normal distribution. For those data sets that were not normally distributed, a Wilcoxon rank sums test was performed to determine whether there was a main effect, followed by pair-wise comparisons. For the data that were normally distributed, an ANOVA test was performed to determine whether there was a main effect, followed by pair-wise comparisons using a Students' t test.

## Results

### Rescue of inhibitory synapse strength by GABA_A_ receptor activation

**Figure 1:**
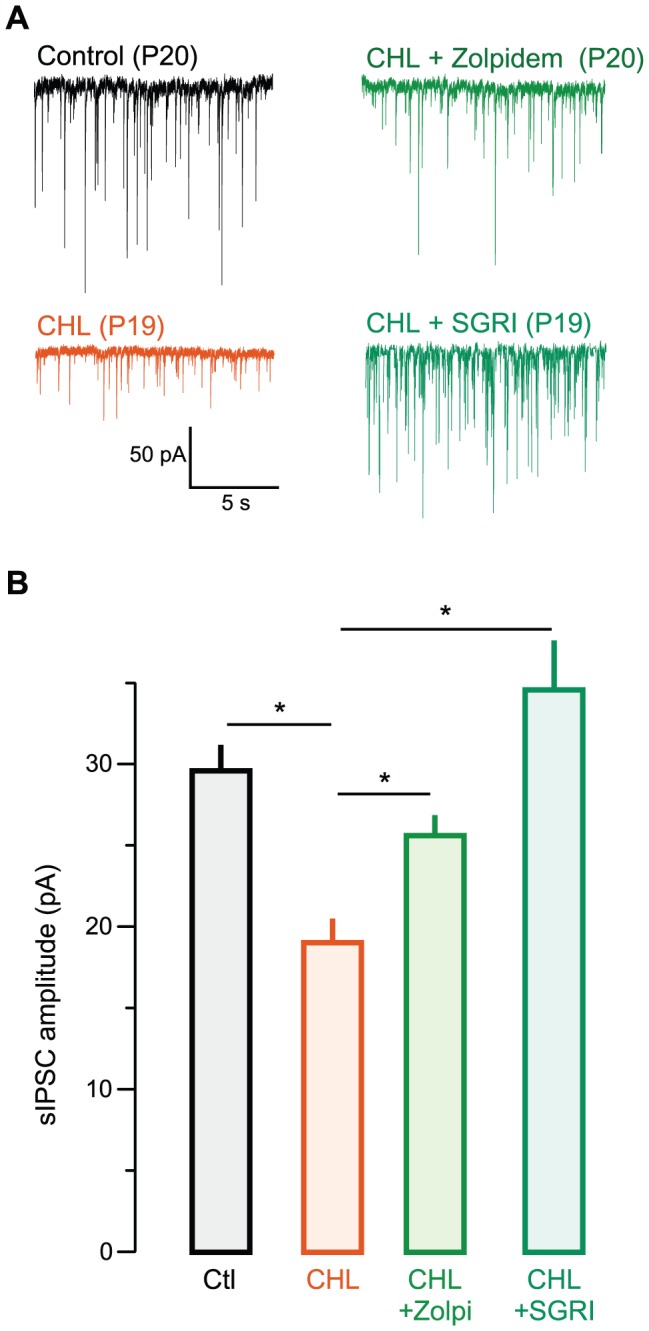
Zolpidem or SGRI each rescue sIPSC strength in juveniles following P10 hearing loss. **A** Representative spontaneous (s) IPSCs recorded in layers 2/3 (L2/3) pyramidal cells in ACx in slices from animals aged postnatal day (P) 18 to P22 (holding voltage, V_HOLD_ = −60 mV). Examples are shown from control (top left, black), CHL (bottom left, orange), zolpidem-treated CHL (top right, green), and SGRI-treated (bottom right, forest green) animals. **B** Bar graph (mean ± SEM) of average amplitude from all recorded neurons showing diminished sIPSC amplitude in CHL animals, and restoration of inhibitory strength after treatment with zolpidem or SGRI.

We first asked whether administration of a GABA_A_ receptor agonist, zolpidem, could rescue inhibitory synaptic strength in juvenile animals reared with CHL (Fig. 1). As reported previously [14], neurons recorded from juvenile animals with CHL displayed significantly smaller sIPSCs compared to age-matched controls. (mean pA ± SEM; Control: 29.9±3.1, n = 19 (11 animals) vs. CHL: 18.7±2.4, n = 10 (7 animals), χ^2^ = 6.3, p = 0.01; Fig. 1). This finding was reconfirmed by comparing animals with sham surgery to a new group of animals with CHL surgery at P10 (see Suppl. Fig. S1 *A*). When zolpidem was administered for the 7 days immediately following CHL at P10, sIPSCs were significantly larger than those from untreated CHL animals [CHL: 18.7±2.4, n = 10 (7 animals) vs. zolpidem-treated CHL: 25.2±2.2; n = 8 (5 animals), χ^2^ = 4.1, p = 0.04; Fig. 1]. There was no difference between control and zolpidem-treated CHL animals, indicating restoration of inhibitory strength (mean pA ± SEM; Control: 29.9±4.0, n = 19 vs. zolpidem-treated CHL: 25.2±1.4; n = 8, χ^2^ = 0.1, p = 0.95; animal number as above). Furthermore, there was no effect of sham injections in CHL animals (see Fig. S1).

**Figure 2:**
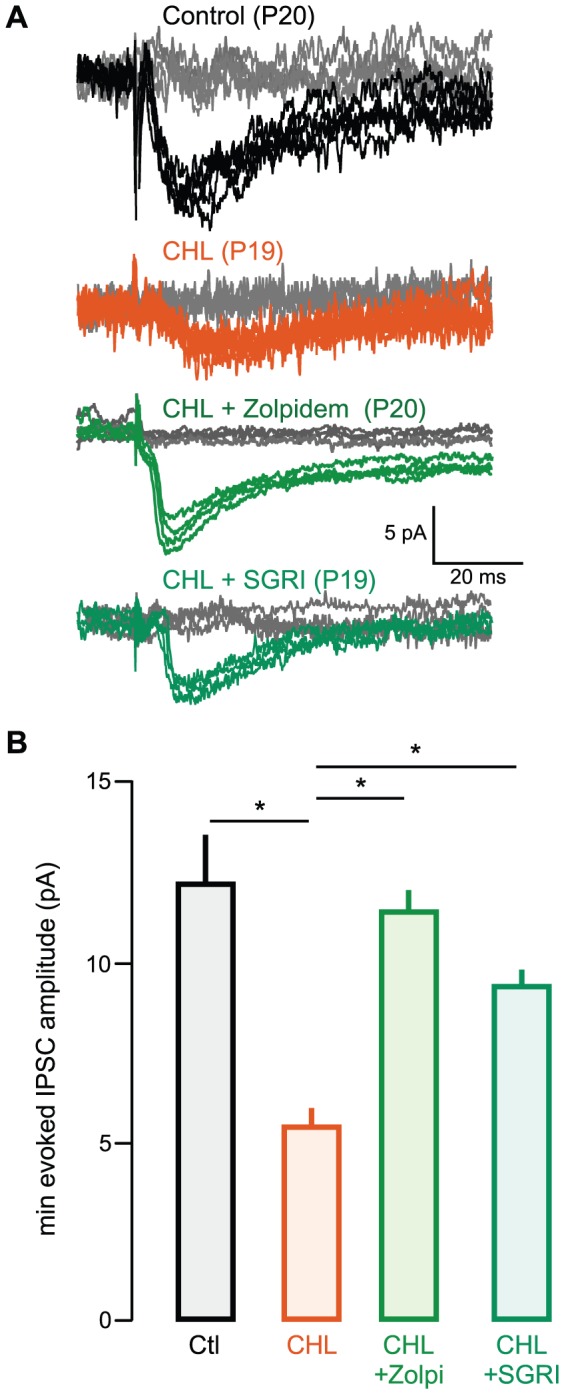
Rescue of inhibitory strength is evident for minimum evoked IPSCs. **A** Representative traces of minimum-amplitude evoked IPSCs (me-IPSCs) recorded in L2/3 pyramidal cells in ACx in slices from animals aged postnatal day (P) 18 to P22 (V_HOLD = _−60 mV). Examples are shown from control (top, black), CHL (second trace, orange). Zolpidem-treated CHL (third trace, green), and SGRI-treated CHL (bottom, forest green) animals. In each of these panels, the gray traces represent failures at sub-minimal stimulus intensities. **B** Bar graph (mean ± SEM) of average me-evoked IPSC amplitude from all recorded neurons showing diminished me-IPSC amplitude in CHL animals is rescued by treatment with zolpidem or SGRI.

Figure 2 shows that minimum evoked (me) IPSCs were significantly smaller in CHL neurons than those from controls [mean pA ± SEM; control: 12.1±1.3, n = 9 (4 animals) vs. CHL: 5.3±0.5, n = 8 (4 animals); t = 4.7, p = 0.0007). In contrast, me-IPSCs recorded in zolpidem-treated CHL animals were significantly larger than those recorded in untreated CHLs (CHL: 5.3±0.5, n = 8 vs. zolpidem-treated CHL: 11.3±1, n = 8 (4 animals), t = 5.1, p = 0.0001). There was no difference between me-IPSCs amplitude recorded in control and zolpidem-treated CHLs (control: 12.1±1.3, n = 9 vs. zolpidem-treated CHL: 11.3±0.5, n = 8, t = 0.4, p = 0.63).

The third measure of inhibitory function was an analysis of sIPSCs frequency. Consistent with our published data [14], CHL did not produce any significant effect on sIPCS frequency (mean Hz ± SEM, control: 3.5±0.6, n = 19 vs. CHL: 4.1±0.6, n = 10, *X^2^* = 1.9, p = 0.17, animal numbers as in sIPSC amplitudes above).

To test for potential confounds introduced by our manipulations, we examined inhibitory function in animals with sham surgery, and in CHL animals given sham injections. Pair-wise comparisons for sIPSCs amplitude showed that sham surgery had no effect on sIPSC amplitude, min-evoked IPSC amplitude, or paired-pulse ratios (Fig. S1). Similarly, sham-injected CHL animals displayed no significant differences from CHLs in these measures (Fig. S1). Consistent with our published data [12], pair-wise comparisons showed that the PPR had shifted from facilitation to depression (mean PPR ± SEM; sham-surgery control: 2.9±1.4 n = 7 (3 animals) vs. CHL: 0.86±0.08, n = 6 (3 animals), *X^2^* = 6.6, p = 0.01).

### Rescue of inhibitory synapse strength by a GABA reuptake inhibitor

We next asked whether administration of a selective GABA reuptake inhibitor (SGRI) could preserve inhibitory synaptic strength in juvenile animals reared with CHL. As shown in Figure 1, SGRI was also able to rescue sIPSC amplitude when administered to CHL animals for 7 days [mean pA ± SEM; CHL: 18.7±2.4 n = 10 (7 animals) vs. SGRI-treated CHL: 34.3±6.5; n = 11 (7 animals), χ^2^ = 4.5, p = 0.03]. Furthermore, there was no difference between sIPSCs from control and SGRI-treated CHL animals (Control: 29.9±3.1, n = 19 vs. SGRI-administered CHL: 34.3±6.5; n = 11, χ^2^ = 0.005, p = 0.95). SGRI administration in CHL animals also restored me-IPSCs amplitudes [CHL: 5.3±0.5, n = 8 (3 animals) vs. SGRI-treated CHL: 9.4±0.5; n = 7 (3 animals), χ^2^ = 10, p = 001]. There was no difference between me-IPSCs from control and SGRI-treated CHLs [Control: 12.1±1.3, n = 9 (4 animals) vs. SGRI-treated CHL: 9.4±0.6; n = 7 (3 animals), χ^2^ = 2.3, p = 0.1]. In contrast to these results, when animals with sham surgery were treated with SGRI, there was no significant change in either sIPSC amplitude or PPR (Fig. S1).

### GABA_B_ receptor agonist did not rescue inhibitory strength

**Figure 3:**
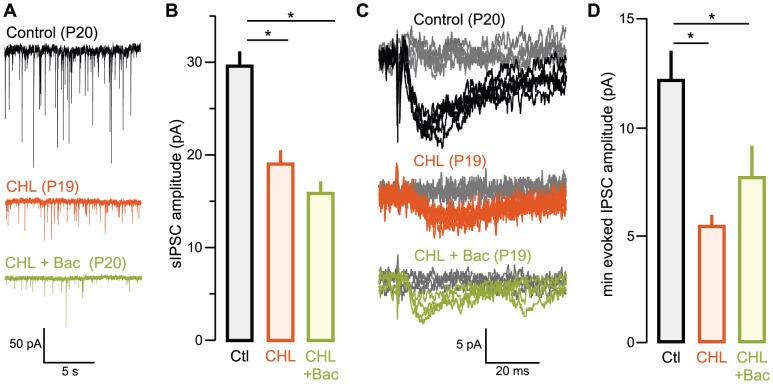
Baclofen does not rescue inhibitory strength in juveniles after P10 hearing loss. **A** Representative sIPSCs recorded in L2/3 pyramidal cells in ACx in slices from animals aged P18 to P22 (V_HOLD_ = −60 mV). Examples are shown from control (top, black), CHL (middle, orange), and BAC-treated CHL (bottom, olive) animals. **B** Bar graph (mean ± SEM) of average sIPSC amplitude from all recorded neurons showing diminished sIPSC amplitude in CHL animals is not rescued after treatment with BAC. **C** Representative traces of minimum-amplitude evoked IPSCs (me-IPSCs) recorded in L2/3 pyramidal cells in ACx in slices from animals aged postnatal day (P) 18 to P22 (V_HOLD = _−60 mV). Examples are shown from control (top, black), CHL (middle, orange) and BAC-treated CHL (bottom, olive). In each of these panels, the gray traces represent failures at sub-minimal stimulus intensities. **D** Bar graph (mean ± SEM) of average me-evoked IPSC amplitude from all recorded neurons showing diminished me-IPSC amplitude in CHL animals is not rescued by treatment with BAC.

In the third set of experiments, we asked whether a GABA_B_ receptor agonist, could restore inhibitory synapse function in juvenile animals reared with CHL (Fig. 3). Here, we found that BAC did not rescue either sIPSC amplitudes when administered to CHL animals [mean pA ± SEM: CHL: 18.7±2.4, n = 10 (7 animals) vs. BAC-treated CHL: 15.5±1.8; n = 11 (7 animals), χ^2^ = 1.1, p = 0.3; control: 29.9±3.1, n = 19 (11 animals) vs. BAC-treated CHL: 15.5±1.8; n = 11 (8 animals), χ^2^ = 12.02, p = 0.0006), or me-evoked IPSC amplitudes [mean pA ± SEM: CHL: 5.3±0.5, n = 8 (4 animals) vs. BAC-treated CHL: 7.6±1.8, n = 7 (3 animals), χ^2^ = 0.9, p = 0.4].

### Rescue of inhibitory synapse strength persisted in adulthood

CHL induced at P10 leads to a persistent reduction of sIPSC amplitude recorded in adults at P90–110 [14]. Therefore, we asked whether pharmacological reinstatement of inhibitory currents observed in juveniles persisted into adulthood. CHL was induced at P10, and each of the three GABAergic agents was administered to separate groups of CHL animals from P11–17. Animals were weaned at P30, reared until adulthood, and recordings were obtained from brain slices at P90–110.

**Figure 4:**
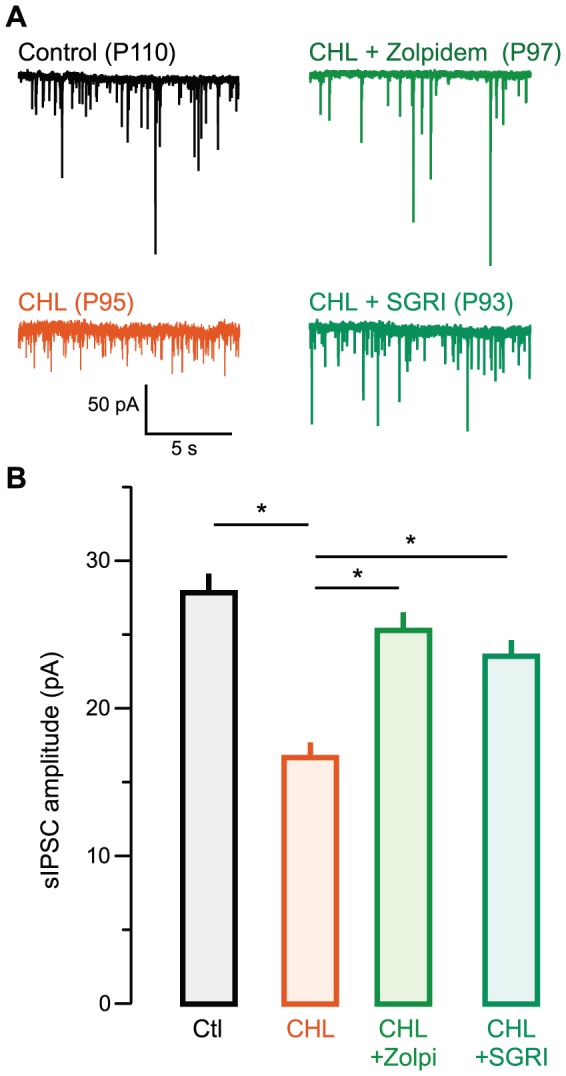
Rescue of inhibitory strength by zolpidem or SGRI persists in adults. **A** Representative sIPSCs recorded in L2/3 pyramidal cells in ACx in slices from animals aged postnatal day (P) 90 to 110 (V_HOLD_ = −60 mV). Examples are shown from control (top left, black), CHL (bottom left, orange), zolpidem-treated CHL (top right, green), and SGRI-treated (bottom right, forest green) animals. **B** Bar graph (mean ± SEM) of average amplitude from all recorded neurons showing diminished sIPSC amplitude in CHL animals, and restoration of inhibitory strength after P11–17 treatment with zolpidem or SGRI.

CHL induced at P10 resulted in a persistent reduction of sIPSC amplitude when recorded in adults at P90–110 (mean IPSC pA ± SEM; Control adult: 27.2±2.7, n = 29 (16 animals) vs. CHL adult: 16.5±2.1, n = 11 (6 animals), χ^2^ = 8.2, p = 0.004) [14]. As shown in Figure 4, the outcome of drug-treatment, when tested in adulthood, was consistent with observations made in juveniles. Administration of either zolpidem or SGRI, but not BAC, preserved sIPSC amplitudes in adult animals reared with CHL [mean pA ± SEM; CHL adult: 16.5±2.1 pA, n = 11 (6 animals) vs. zolpidem-treated CHL adult: 25.6±2.4 pA; n = 9 (6 animals), χ^2 = ^ = 5.7, p = 0.01]. There was no difference between sIPSCs from adult controls and zolpidem-treated CHL adults (Control: 27.2±2.1, n = 29 vs. zolpidem-treated CHL adult: 25.6±2.4; n = 9, χ^2^ = 0.3, p = 0.7).

The effect of SGRI administration during juvenile development also persisted when sIPSCs were recorded in adult animals [Fig. 4; mean pA ± SEM; CHL adult: 16.5±2.1 pA, n = 11 (7 animals) vs. SGRI-treated CHL adult: 23.6±1.4 pA; n = 6 (4 animals), χ^2^ = 3.6, p = 0.05]. There was no difference between sIPSCs from control and SGRI-treated CHL adults [mean pA ± SEM; Control adult: 27.2±2.7, n = 29 (16 animals) vs. SGRI-treated CHL adult: 23.6±1.4; n = 6 (4 animals), χ^2^ = 0.01, p = 0.89). In contrast, BAC-treated CHL animals continued to display IPSCs resembling those of CHL animals when assessed in adulthood (Fig. S3), indicating an absence of restoration [mean pA ± SEM; CHL adult: 16.5±2.1, n = 11 (7 animals) vs. BAC-treated CHL adult: 18.5±1.2 pA; n = 8 (χ^2^ = 1.5, p = 0.21; Control adult: 27.2±2.7, n = 29 vs. BAC-treated CHL adult: 18.5±1.1; n = 8 (4 animals), χ^2^ = 3.6, p = 0.05).

### Rescue of inhibitory strength occurred following bilateral cochlear ablation

**Figure 5:**
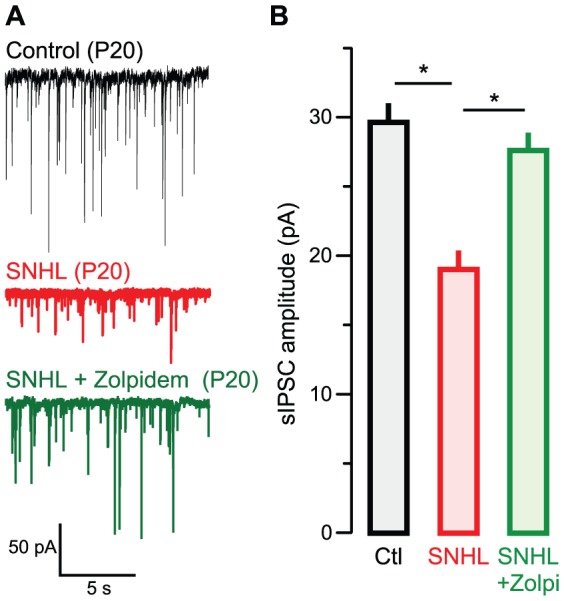
Zolpidem rescues inhibitory strength following cochlear ablations. **A** Representative sIPSCs recorded in L2/3 pyramidal cells in ACx in slices from animals aged postnatal day P18–22 (V_HOLD = _−60 mV). Example traces are shown from control (top, black), SNHL (middle, red), and Zolpidem-treated (bottom, green) animals. **B** Bar graph (mean ± SEM) of average sIPSC amplitude from all recorded neurons showing diminished sIPSC amplitude in SNHL animals is rescued by treatment with Zolpidem.

To determine whether it is possible to preserve inhibitory strength even after complete loss of auditory afferents, we performed bilateral cochlear ablations at P10, followed by administration of zolpidem from P11–17. Zolpidem rescued sIPSC amplitudes in cochlear-ablated animals, just as it did in CHL animals. Figure 5 shows that neurons recorded from juvenile animals reared with cochlear ablations displayed significantly smaller sIPSCs [mean pA ± SEM; Control: 29.9±3.1, n = 19 (11 animals) vs. ablated: 18.4±2.4, n = 12 (7 animals), χ^2^ = 9.9, p = 0.001). However, sIPSCs were significantly larger in zolpidem-treated cochlear ablated animals as compared to untreated ablated animals (ablated: 18.4±2.4, n = 12 vs. zolpidem-treated ablated: 27.2±2.4; n = 10 (6 animals), χ^2^ = 5.3; p = 0.02). There was no difference between sIPSC amplitude between control and zolpidem-administered ablated animals (Control: 29.9±3.1, n = 19 vs. zolpidem-treated ablated: 27.2±2.4; n = 10, χ^2^ = 0.008, p = 0.92).

### Rescue of inhibitory strength was observed after delayed SGRI administration

**Figure 6:**
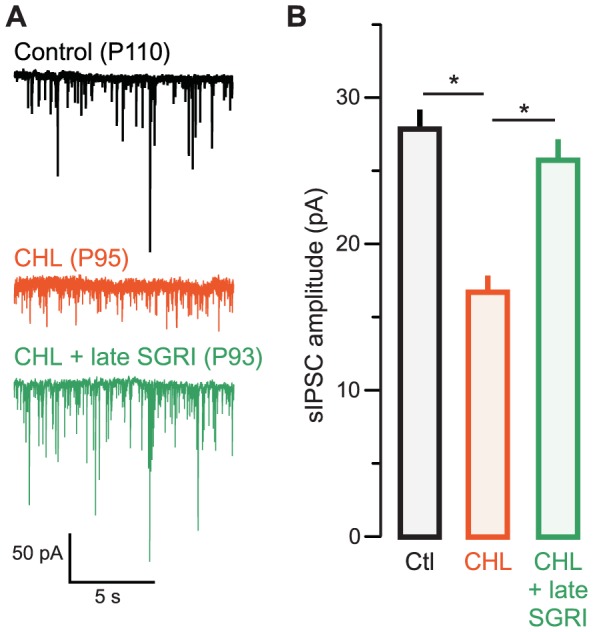
Delayed SGRI intervention also rescues inhibitory strength in adults. **A** Representative sIPSCs recorded in L2/3 pyramidal cells in ACx in slices from animals aged P90–110 (V_HOLD = _−60 mV). Examples are shown from control (top, black), CHL (middle, orange), and SGRI-treated (bottom, forest green) animals. **B** Bar graph (mean ± SEM) of average sIPSC amplitude from all recorded neurons showing diminished sIPSC amplitude in adult CHL animals is rescued by P30–36 SGRI administration.

To determine whether GABA receptor activation could preserve IPSC strength when treatment was delayed by about 3 weeks from CHL induction, we induced CHL at P10, administered SGRI at P30–36, and raised animals to adulthood. As shown in Figure 6, SGRI could restore sIPSC amplitudes in adults when treatment was delayed [mean pA ± SEM; adult control: 27.2±2.7, n = 29 (16 animals) vs. SGRI-treated at 30–36 CHL adult: 26.8±3.7; n = 9 (6 animals), χ^2 = ^0.04, p = 0.82; CHL adult: 16.5±2.1, n = 11 vs. SGRI-treated P30–36 CHL adult: 26.8±3.7; n = 9 χ^2^ = 5.75, p = 0.01].

## Discussion

Developmental hearing loss may originate in the periphery, yet it causes pervasive impairment of CNS synapses and membrane properties. The disruption of one such property, inhibitory synaptic strength, has been suggested to be a causative factor in many central disorders, including hearing loss [10], [11], [12], [13], [26], [27], [28], [29], [30], [31]. In fact, developmental hearing loss results in weakened inhibitory synapse strength that persists into adulthood [14]. Our findings suggests that *in vivo* GABA_A_ receptor activation is sufficient to promote the maturation of inhibitory synaptic strength in CHL animals, and this principle may apply to other disorders associated with diminished inhibition.

### Activity-dependent regulation of developing GABAergic synapses

Converging lines of evidence indicate that inhibitory synapse maturation is facilitated by both spontaneous and driven activity [2], [4], [8]. In the auditory system, disruption of normal activity can perturb both inhibitory synapse strength and the specificity of anatomical projections [32], [33], [34], [35], [36], [37]. Similarly, it has been found that normal activity is necessary for the maturation and maintenance of inhibitory synaptic currents in sensory cortices and dissociated cultures [38], [39], [40], [41], [42], [43], [44], [45]. Consistent with this principle, our results demonstrate that activation of the GABA_A_ receptor a1 subunit (via zolpidem) or an increase in extracellular GABA at inhibitory synapses (via SGRI action) could each preserve sIPSC amplitude in juvenile animals reared with CHL (Figs. 1, 2). Furthermore, inhibitory strength remained comparable to control levels in adult CHL animals long after drug treatment was terminated (Fig. 4). This finding is broadly consistent with the result that loss of GABA synthesis leads to a reduction of cortical inhibitory boutons and GABAergic transmission, and this can be reversed by activating GABA_A_ receptors [16], [46].

### Temporal window for rescue of inhibitory strength

Descriptive findings from the rodent auditory CNS indicate that many inhibitory synapse properties reach adult-like characteristics within the first 2–3 postnatal weeks [12], [47], [48], [49], [50], [51], [52], [53], [54]. Therefore, we expected that GABAergic activation would no longer rescue inhibitory strength if it were initiated after 4 postnatal weeks. Contrary to this prediction, we found that the time window during which GABA_A_ receptor activation is sufficient to restore inhibitory strength after CHL extended beyond one month (Fig. 6). Therefore, cortical inhibitory synaptic strength remains sensitive to environmental perturbation after the age when it would normally have reached maturity.

This result is consistent with findings from the visual system suggesting that deprivation extends the critical period. For example, when kittens are reared in the dark, it is found that monocular deprivation can continue to induce changes in cortical processing in adult animals [55]. Similarly, in the auditory system, the sensitive period can be extended by rearing animals in a pulsed noise environment [56]. Our results are consistent with a delay in the closure of a sensitive period for inhibitory synaptic maturation. Thus, for animals reared with CHL, we found that late administration of SGRI was able to restore inhibitory strength (Fig. 6). Therefore, our finding indicates that the auditory deprivation due to CHL extended the critical period such that SGRI could induce a positive effect.

### Possible mechanism for restoration of inhibitory strength

Injury or inactivation of the cochlea leads to reduced central spontaneous activity during development and into adulthood [57], [58], [59]. Therefore, the enhancement in excitation and concomitant decrease in inhibition following severe hearing loss [10] is consistent with a homeostatic response to the decrease in postsynaptic activity [60], [61]. However, if a purely homeostatic mechanism was operative, then GABA_A_ agonist treatment should not have resulted in more inhibition. Thus, diminished activity does not provide a complete explanation of the outcome. Since these results suggest a non-homeostatic mechanism, it is possible that developing inhibitory synapses display unique responses to manipulations of activity. For example, hearing loss reduces the trafficking of GABA_A_ subunits into ACx inhibitory synapses [62], and it is possible that GABA_A_ activation restores this process. Agonist-mediated activation of the inhibitory postsynaptic anchoring protein, gephyrin, can facilitate clustering and restore the GABA_A_ receptor accumulation [63], [64], [65]. Other GABA_A_ receptor trafficking mechanisms could operate in synergy with gephyrin to normalize receptor trafficking and restore inhibitory strength [63], [64], [65], [66], [67], [68], [69]. In fact, sustained activation of GABA_A_ receptors by its potent agonist, muscimol, is also known to facilitate IPSCs via increased trafficking of GABA_A_ receptors to the postsynaptic membrane [70]. Since the artificial cerebrospinal fluid (ACSF) and internal pipette solution were identical for all groups of animals, it is unlikely that chloride transport mechanisms [71] contribute to our observations.

We have provided evidence that preservation of cortical inhibitory synaptic strength is feasible in animals with moderate or severe hearing loss. Since we do not know the effect of GABA_A_ agonists on excitatory synapses [72] or voltage-gated channels, it is premature to infer that cortical networks will be fully restored following the pharmacological procedures employed here. However, the impact of GABAergic activation does persist long after treatment ceases (Figs. 4, and 6), suggesting that the potential ameliorative effect of pharmacological treatment can be tested behaviorally [73].

## Supporting Information

Figure S1 **Bar graph representation of sIPSC amplitude (mean pA ± SEM, top panel, A) and paired pulse ratio of evoked IPSCs (bottom panel).** n values appear within bars. The sIPSC amplitudes in sham-surgery animals were significantly different from CHL and CHL with sham injection groups (asterisk). The PPR was facilitatory in sham-surgery animals and depressing in the remainder group. See S1 Results for pair-wise comparisons.
(EPS)

Figure S2 **The scatter plot shows the distribution of all sIPSC amplitudes recorded from 4 experimental groups.**
(EPS)

Figure S3 **Inability of BAC to rescue inhibition persists in adults.** **A** Left panel: Representative sIPSCs recorded in L2/3 pyramidal cells in ACx in slices from animals aged P90–110 (V_HOLD = _−60 mV). Examples are shown from control (top, black), CHL (middle, orange) and BAC-treated CHL (bottom, olive) animals. **B** Right panel: Bar graphs (mean ± SEM) of average sIPSC amplitude from all recorded neurons showing diminished sIPSC amplitude in CHL animals is not rescued after treatment with BAC.
(EPS)

Results S1 (DOC)

Methods S1 (DOC)
