# Supplementary material for: Rescue of Inhibitory Synapse Strength following Developmental Hearing Loss
Source: PLoS One. 2013 Jan 11;8(1):e53438. doi: 10.1371/journal.pone.0053438 (PMC3543446; doi:10.1371/journal.pone.0053438)
Supplement: Results S1 — (DOC) [file pone.0053438.s004.doc]

**Supporting Information**

**S1 Results**

Although each pair-wise comparison is provided in the Results, here we present the test for normal distribution with mean ± SEM for each measure. The sIPSC amplitudes recorded from juveniles were not normally distributed in each of the 7 groups: control, CHL, zolpidem-treated CHL, SGRI-treated CHL, BAC-treated CHL, SNHL, and zolpidem-treated SNHL (see Fig. S2). There was a main effect of treatment group (n=81, χ^2^=26, df=6, p=0.0001), and the mean sIPSC amplitudes for each group were as follows (pA ± SEM): control: 29.9 ± 3.1, n=19 (11 animals), CHL: 18.7 ± 2.4, n=10 (7 animals); zolpidem-treated CHL: 25.2 ± 2.2; n=8 (5 animals); SGRI-treated CHL, 34.3 ±6.5; n=11 (7 animals); BAC-treated CHL: 15.5 ± 1.8; n=11 (8 animals); SNHL: 18.4 ± 2.4 pA, n=12 (7 animals); zolpidem-treated SNHL: 27.2 ± 2.4 pA; n=10 (6 animals).

The effect of GABAergic agent administration was also assessed by measuring the minimum-evoked (me) IPSC amplitudes elicited by minimum stimulation to L4 (i.e., putative unitary inhibitory connections) onto pyramidal neurons in juveniles. There was a main effect of treatment group (ANOVA, n=38, F=6, p=0.008), and the me-IPSCs distribution was not normal. The mean me-IPSC amplitudes for each group were as follows (pA ± SEM): control: 12.1 ± 1.3, n=9 (4 animals) vs. CHL: 5.3 ± 0.5, n=8 (4 animals), zolpidem-treated CHL: 11.3 ± 1, n=8 (4 animals); SGRI-treated, 9.4 ± 1.2, n=7; BAC-treated CHL: 7.6 ± 1.8, n=7 (3 animals).

The sIPSC amplitudes recorded from animals that received either a sham surgery or sham injection were not normally distributed among the 4 groups; sham-surgery control, CHL, sham-injected CHL, SGRI-administered sham surgery animals, sIPSC amplitude, mean pA ± SEM; sham-surgery control: 27.2 ± 2.8, n=9 (4 animals), CHL: 16.2 ± 0.9, n=9 (5 animals); vehicle-treated CHL: 17.8 ± 0.8, n=8 (4 animals); SGRI-treated sham-surgery: 22.6 ± 2.4, n=7 (3 animals); χ^2^=11; df=3; p=0.01 (n=31). Figure S1 (top panel) shows bar graph representation of these data. me-IPSC amplitude: mean pA ± SEM; sham-surgery: 10.6 ± 0.8, n=9; CHL: 5.5 ± 0.3, n=6; sham-injected CHL: 5.7 ± 0.3, n=8; SGRI-treated sham-surgery: 11.1 ± 0.7, n=7; χ^2^=21.5; df=3; p=0.0001, (n=31, Breakdown of animal number is same as above). Paired-pulse ratio (PPR, pA IPSC2/IPSC2, P2/P1); mean ± SEM; sham-surgery: 2.9 ± 1.4, n=7; CHL: 086 ± 0.08, n=6, sham-injected CHL: 0.8 ± 0.04, n=8; SGRI-treated sham-surgery: 1 ± 0.1; n=8; χ*^2^*=11.3; df=4; p=0.02 (n=29; breakdown of animal number as above except we did could not record PPR in 2 animals). Figure S1 shows bar graph representation of the PPR data. See results for pair-wise comparisons.

The sIPSC amplitudes recorded from adults were not normally distributed among the 7 groups of animals: control adult (P90-110), control P30-36, CHL adult (CHL performed at P10), zolpidem-treated CHL adult, SGRI-treated (at P30-36) CHL adult, and BAC-treated CHL adult. There was a main effect of treatment group (n=80, χ^2^=15.74; df=6; p=0.01) and the mean sIPSC amplitudes for each group were as follows (pA ± SEM) control adult: 27.2 ± 2.8, n=19 (9 animals), control P30-36, 27.2 ± 2.4, n=8 (4 animals); CHL adult: 16.5 ± 2.1, n=11 (6 animals); zolpidem-treated CHL adult: 25.6 ± 2.4; n=9 (5 animals); SGRI-treated CHL adult: 23.6 ± 1.4; n=6 (3 animals); BAC-treated CHL adult: 18.5 ± 1.1; n=8 (4 animals).
