# Supplementary material for: Rescue of Inhibitory Synapse Strength following Developmental Hearing Loss
Source: PLoS One. 2013 Jan 11;8(1):e53438. doi: 10.1371/journal.pone.0053438 (PMC3543446; doi:10.1371/journal.pone.0053438)
Supplement: Methods S1 — (DOC) [file pone.0053438.s005.doc]

**Supporting Information**

**S1 Methods**

The data represent whole-cell voltage-clamp recordings from 205 supragranular pyramidal neurons in thalamorecipient ACx. Of these, 62 recordings were from P10 CHL surgery recorded at P18-23, adult controls recorded at 90-110, and P10 CHL surgery recorded at P90-110 appear in a recent report [14]. Thus, data from 143 recordings are new, including 31 recordings from a new P10 CHL group, a sham surgery group, a sham injection group, an SGRI-injected sham surgery group, and pilot CHL group in which suboptimal dose of GABA agonists did not restore IPSCs. Recordings were obtained from neurons at P18-23 (juveniles) or P90-110 (adults). The number of neurons and animals for each set of experiment was: control recorded at P18-23, n=19 (11 animals); control recorded at P90-100, n=29 (16 animals); P10 sham-surgery, n=6 (4 animals); P10 CHL recorded at P18-23, n=10 (7 animals); P10 CHL recorded at P90-110, n=11 (6 animals); vehicle-injected P10 CHL recorded at P18-23, n=6 (4 animals); P10 bilateral cochlear ablation recorded at P18-23, n=12 (7 animals); zolpidem-injected P10 CHL recorded at P18-23, n=8 (5 animals); SGRI-injected P10 CHL recorded at P18-23, n=11 (7 animals); BAC-injected P10 CHL recorded at P18-23, n=11 (8 animals); zolpidem-injected P10 CHL recorded at P90-110, n=9 (6 animals); SGRI-injected P10 CHL recorded at P90-110, n=6 (4 animals); BAC-injected P10 CHL recorded at P90-110, n=8 (4 animals); zolpidem-injected P10 bilateral cochlear ablation recorded at P18-23, n=10 (6 animals); SGRI-injected sham-surgery recorded at P18-23, n=7 (4 animals); SGRI late-injected P10 CHL recorded at P18-23, n=9 (4 animals), and a CHL group in which 1mg/kg of the 3 agents were administered in 2 pups each, n=6 (6 animals). We did not statistically test differences in sIPSC amplitudes in CHL animals administrated with 1mg/kg doses of zolpidem, n=2 (2 animals)., SGRI, n=2 (2 animals) and BAC, n=2 (2 animals) although their averages were compared with sIPSCs recorded in CHL animals.

Spontaneous (s) and minimum evoked (me) IPSC amplitudes were analyzed in all juvenile and adult animals, as described previously [11]. The sIPSCs magnitude considered only events above 8 pA. To examine whether the strength of putative monosynaptic inhibitory connections was altered following SNHL, me-IPSCs were recorded in normal ACSF in voltage clamp conditions at V_HOLD_=-60 mV. Incremental stimulus intensities were delivered to layer 4 at 0.1 Hz until an evoked IPSC was observed. Stimulation at this intensity produced ≥ 50% failures, and this stimulation intensity was kept constant throughout each recording. We did not directly compare the amplitudes of the spontaneous IPSCs with the me-IPSCs, although the strength of both was significantly reduced following CHL.

To determine whether rescue was feasible if an agonist was administered several weeks after CHL, SGRI was administered in CHL animals (surgery at P10) at P30-36 and sIPSCs subsequently recorded in adults. To determine whether rescue was feasible after severe hearing loss, sIPSCs were recorded in zolpidem-administered pups in juveniles (Supplemental Table 1).

**Animals:** All experiments were performed on gerbils (*Meriones unguiculatus*) aged postnatal day (P) 10-110 that were born in our animal facility from breeding pairs (Charles River). The data are drawn from voltage-clamp recordings in 199 supragranular pyramidal neurons, each recorded in a separate thalamorecipient ACx slice preparation.

**Conductive hearing loss surgery:** Gerbil pups at P10 were anesthetized with halogenated ethyl methyl ether methoxyflurane (Anafane, Medical Developments International Ltd.) and anesthetic induction occurred within 10 minutes, which was confirmed by a complete elimination of responses to nociceptive stimuli. For adult surgery, animals were anesthetized with isoflurane. A post-auricular skin incision was made, and the tympanic membrane was visualized and punctured. The malleus was then extirpated through this opening. The postauricular wound was closed with cyanoacrylate glue, and the procedure repeated on the other side. After surgery, animals were warmed on a heating pad and returned to the litter when respiration and motor activity had recovered. Animals were reared for 8-13 days under conditions identical to those for control pups. In some experiments, animals were reared for 20-34 days or 80-90 days after surgery at P10. When brain slices were prepared, the stapes was visualized to ensure stability at the oval window. Malleus removal did not disrupt the stapes in any of the animals used for this study. Sham surgery was performed at P10. Each animal was anesthetized, an incision was made in the skin over each ear canal, the wound was closed, and the animal was then allowed to recover.

**Cochlear ablation surgery:** Gerbil pups were anesthetized (see above), and each cochlea was rapidly removed with forceps. Animals were reared 8-13 days under conditions identical to those for control pups. After generating brain slices, cochlea removal was confirmed post mortem by opening the inner wall of the cochlea and observing the absence of cochlear tissue and the presence of a Gelfoam insert. Slices were discarded when a partial or an incomplete removal was observed.

**Drug treatment:** A caveat associated with subcutaneous drug injections is that they can may systemically affect the developing central and peripheral nervous system. However, there are also advantages for subcutaneous administration: (i) daily delivery of agonist was relatively non-invasive, (ii) delivery of agonists in the skin was rapid, (iii) SC administration circumvented the additional use of surgical anesthetic that could affect inhibitory synapse development, (iv) locomotor and feeding behavior was unimpeded, except in case of 10/mg BAC that was discontinued, and (v) each agonist could cross the blood brain barrier.

**Control manipulations:** Several experiments were performed to determine whether surgical procedures, anesthesia, SC injection, or date of data collection had an effect on inhibitory function. IPSCs were examined in brain slices generated from 4 groups of animals: (a) a new set of CHL surgery at P10, (b) Sham surgery at P10, (c) sham injection in CHL animals, and (d) SGRI treatment of P10 sham surgery animals.

For sham surgery performed at P10, each animal was anesthetized (above), an incision was made in the skin over each ear canal, the wound then closed, and the animal was allowed to recover. For sham injections, vehicle alone (water, 0.05 ml/pup, SC) was administered from P11-17 in CHL animals. Since we are comparing data from a recent publication (14), a new group of animals with CHL surgery at P10 was obtained for comparison to the sham-surgery data. To test the effect of GABA agonist, one group of sham-surgery animals were treated with SGRI (10 mg/kg, SC) at P11-17.

**Brain slice preparation:** The brain was sectioned perihorizontally at 15^o^ to preserve the ventral medial geniculate (MGv) and its ascending pathways to the ACx. The slices were incubated in ACSF at 32^o^C for 30-45 minutes, kept at room temperature for an hour, and transferred to a recording chamber continuously superfused (~3mL/min) with ACSF at 32±1^o^C. The ACSF contained (in mM): 125 NaCl, 4 KCl, 1.2 KH_2_PO_4_, 1.3 MgSO_4_, 24 NaHCO_3_, 15 glucose, 2.4 CaCl_2_ and 0.4 L-ascorbic acid (pH 7.3 when bubbled with 95%O_2_/5%CO_2_). Whole cell recording were obtained from the thalamorecipient ACx previously validated by extracellular field activity recorded in response to MGv stimulation. For adult (P90-110) slices, the slice thickness was reduced to 300 µm to enhance viability and visualized recordings, while the perihorizontal slice angle was increased to 25^o^. Ionotropic glutamate receptor blockers, 6,7-Dinitroquinoxaline-2,3-dione (DNQX, 20 µM) and 2-amino-5 phosphonopentanoate (AP-5, 50 µM) were continuously bath applied with ACSF to isolate inhibitory currents (11).

Whole-cell recordings (PC-501A; Warner Instruments, Hamden, CT) were obtained from pyramidal neurons in cortical layers (L) 2/3. Recording electrodes were made from borosilicate glass microcapillaries (outer diameter, 1.5mm) with a puller (P-97; Sutter Instruments, Novato, CA). The internal recording solution contained (in mM): 100 KCl, 40 K-gluconate, 8 NaCl, 10 [4-(2-hydroxyethyl)-1-piperazineethanesulfonic free acid] (HEPES), 2 MgCl_2_, 0.1 ethyleneglycol-bis(2-aminoethylether)-N,N,N’,N’-tetraacetic acid (EGTA), 2 adenosine 5’-triphosphate disodium salt (ATP), 0.3 guanosine 5’-triphosphate sodium salt (GTP) and 5 lidocaine derivative QX-314 (pH=7.2 with KOH). The resistance of these electrodes filled with the internal solution was 5-12 MΩ. Access resistances were 15-40 MΩ and were compensated by about 70%. Pyramidal cells were identified by their characteristically shaped cell bodies under infrared differential interface contrast (IR-DIC) optics at 400X. After breaking the ≥1 GΩ membrane patch, it was possible to immediately record a resting potential (<50mv) and an overshooting action potential before spikes were blocked to validate that cells were healthy. Recordings were then made using voltage-clamp (V_HOLD_=-60mV) after 5 minutes. Inhibitory postsynaptic currents (IPSCs) were recorded in the presence of ionotropic glutamate receptor blockers, 6,7-Dinitroquinoxaline-2,3-dione (DNQX; 20μM; Sigma, St. Louis) and 2-amino-5-phosphopentanoate (AP-5; 50μM; Tocris Cookson, Ballwin) that were added to the oxygenated superfusing ACSF.

For evoked IPSCs, responses were recorded 10 min after the continuous application of the DNQX and AP-5. Electrical stimuli delivered via a stimulus isolator (model BSI-9501; Dagan, Minneapolis) to a bipolar stimulating electrode placed on L4. The stimulating electrodes were made from 0.004” diameter teflon-coated platinum wires (A-M systems, Carlsborg, WA) that were inserted into a 2” long double-barrel glass electrode. The exposed tip was 0.002” diameter. The intracortical stimulation duration was kept constant at 100μs. To determine the stimulus magnitude, incremental stimulus intensities were delivered at 0.05Hz until an evoked IPSC was discernible from failures (11). Stimulation at this intensity produced >50% failures in eliciting IPSCs. The absolute stimulus intensity varied about 5% between preparations and specific ages. Increasing the minimal intensity by more than 5% decreased the failure rate and increased the IPSC amplitude and polysynaptic innervations. Therefore, the minimal stimulation intensity was kept constant throughout each recording. The failure rates were not analyzed [11].

**Data Acquisition and Analysis:** Data were filtered at 5 kHz and acquired at a sampling rate of 10 kHz by a custom-designed IGOR (version 4.08; WaveMetrics, Lake Oswego, OR) macro on a Macintosh platform called SLICE (Apple, Cupertino, CA). A second IGOR macro (SLICE analysis) was used for offline analysis. This macro used a semi-automated procedure to identify events, which used a threshold value based on the SD of the acquired trace. A minimum threshold of -8 pA was used to detect sIPSCs (14). All IPSC data are reported as mean pA ± SEM. Details on the acquisition and analysis of sIPSC amplitude and frequency, me-IPSC amplitude, and PPR are described in a recent paper (14).
